# Attribution of NF-κB Activity to CHUK/IKKα-Involved Carcinogenesis

**DOI:** 10.3390/cancers13061411

**Published:** 2021-03-19

**Authors:** Xin Li, Yinling Hu

**Affiliations:** Laboratory of Cancer Immunometabolism, Center for Cancer Research, National Cancer Institute, National Institutes of Health, Frederick, MD 21702, USA; xin.li3@nih.gov

**Keywords:** IKKα, NF-κB, Carcinogenesis

## Abstract

**Simple Summary:**

CHUK/IKKα has emerged as a novel tumor suppressor in several organs of humans and mice. In general, activation of NF-κB promotes inflammation and tumorigenesis. IKKα reduction stimulates inflammatory responses including NF-κB’s targets and NF-κB-independent pathways for tumor promotion. Specific phenomena from genetically-modified mice and human TCGA database show the crosstalk between IKKα and NF-κB although their nature paths for normal organ development and the disease and cancer pathogenesis remains largely under investigation. In this review, we focus on the interplay between IKKα and NF-κB signaling during carcinogenesis. A better understanding of their relationship will provide insight into therapeutic targets of cancer.

**Abstract:**

Studies analyzing human cancer genome sequences and genetically modified mouse models have extensively expanded our understanding of human tumorigenesis, even challenging or reversing the dogma of certain genes as originally characterized by in vitro studies. Inhibitor-κB kinase α (IKKα), which is encoded by the conserved helix-loop-helix ubiquitous kinase (*CHUK*) gene, is first identified as a serine/threonine protein kinase in the inhibitor-κB kinase complex (IKK), which is composed of IKKα, IKKβ, and IKKγ (NEMO). IKK phosphorylates serine residues 32 and 36 of IκBα, a nuclear factor-κB (NF-κB) inhibitor, to induce IκBα protein degradation, resulting in the nuclear translocation of NF-κB dimers that function as transcriptional factors to regulate immunity, infection, lymphoid organ/cell development, cell death/growth, and tumorigenesis. NF-κB and IKK are broadly and differentially expressed in the cells of our body. For a long time, the idea that the IKK complex acts as a direct upstream activator of NF-κB in carcinogenesis has been predominately accepted in the field. Surprisingly, IKKα has emerged as a novel suppressor for skin, lung, esophageal, and nasopharyngeal squamous cell carcinoma, as well as lung and pancreatic adenocarcinoma (ADC). Thus, *Ikkα* loss is a tumor driver in mice. On the other hand, lacking the RANKL/RANK/IKKα pathway impairs mammary gland development and attenuates oncogene- and chemical carcinogen-induced breast and prostate tumorigenesis and metastasis. In general, NF-κB activation leads one of the major inflammatory pathways and stimulates tumorigenesis. Since IKKα and NF-κB play significant roles in human health, revealing the interplay between them greatly benefits the diagnosis, treatment, and prevention of human cancer. In this review, we discuss the intriguing attribution of NF-κB to CHUK/IKKα-involved carcinogenesis.

## 1. Introduction

**Figure 1:**
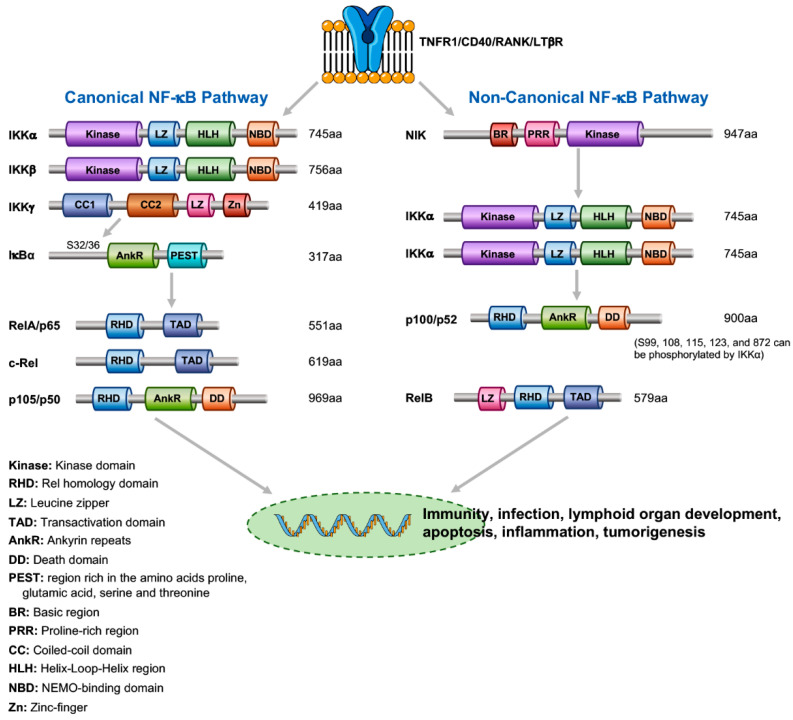
A model of canonical and non-canonical NF-κB pathways. (aa: amino acid; two IKKα molecules form a homodimer in the non-canonical NF-κB pathway).

Five NF-κB transcriptional factors, including RelA (p65), RelB, c-Rel, NF-κB1 (p50), and NF-κB2 (p52), are differentially expressed in a wide range of cell types and execute their physiological functions through the compositions of their homodimers or heterodimers (Figure 1) [1]. Studies using genetically modified mouse models show that the biological roles of these transcription factors partially overlap [2]. These NF-κB components are classified into the canonical and non-canonical NF-κB pathways, as initiated by their upstream regulators [3,4]. In the canonical signaling (Figure 1), the NF-κB inhibitor IκBα interacts with NF-κB p65/p50 dimers that mask the nuclear localization signal of NF-κB, thereby blocking NF-κB’s nuclear translocation from cytosol to nucleus and inactivating NF-κB. In the non-canonical signaling, an NF-κB-inducing kinase (NIK) activates IKKα, which then phosphorylates and then cleaves the p100 to generate p52, resulting in RelB/p52 dimer nuclear translocation [4,5]. The activated NF-κB functions as a transcriptional factor by binding to the DNA consensus sequences on the regulatory elements of the target genes. IKKα and IKKβ, which are highly conserved protein kinases containing a protein kinase domain, a leucine zipper motif, and a helix-loop-helix motif, are catalytical subunits [6,7,8,9,10]. IKKγ is a regulatory subunit in the IKK complex. There is an IKKγ binding motif at the end of the C-terminus of IKKα and IKKβ. Following activation of the receptors, the activated IKK stimulates canonical and non-canonical NF-κB activation and transduces signals from external cells to the cellular nucleus. These IKK subunits are not functionally redundant because *Ikkα^−/−^*, *Ikkβ^−/−^*, or *Ikkγ^−/−^* mice display severe phenotypes (Table 1), although *Ikkβ^−/−^*, *Ikkγ^−/−^*, and *p65^−/−^* mice show a similar phenotype of embryonic lethality from hemorrhage mediated by liver cell death and are rescued by *Tnfr1* deletion, which blocks cell death [11,12,13,14,15,16,17]. *Ikkα^−/−^* mice die within 15 minutes after birth due to water loss from severely impaired skin, and *Tnfr1* deletion does not rescue them [18]. Thus, IKKα plays a distinct physiological function from IKKβ and IKKγ during embryonic development.

Following ligand stimulation, activated receptors on cell surfaces transduce signals from external cells to the cellular cytosol and nucleus, leading to the activation or suppression of specific pathways and events at various levels in the cells. TNFR family members, such as TNFR1/TNF, LTβR/LTβ, BAFFR/BAFF, CD40/CD40L, and RANK/RANKL, which are differentially expressed in a broad variety of specific cells, execute distinct biological activities and regulate tumorigenesis. In general, increased NF-κB activity promotes tumorigenesis. For example, defective RANKL/RANK/IKKα signaling impairs mammary gland development and damps oncogene-mediated breast carcinogenesis [34]. IKKα loss, meanwhile, promotes the development of cutaneous, lung, and esophageal squamous cell carcinomas (SCCs) and lung and pancreatic ADCs [19,22,23,35,36,37,38,39,40], which are associated with increased NF-κB related targets and inflammation. However, the way in which IKKα crosstalks with the NF-κB pathway remains to be fully elucidated. Here, we discuss the connections between IKKα and NF-κB activity in skin, lung, and esophageal SCCs; lung and pancreatic ADCs; and breast and prostate cancer.

## 2. IKKα, NF-κB, and Carcinogenesis

### 2.1. Squamous Cell Carcinoma

The surfaces of our bodies, some internal organs, and the interface of the tracts, which connect or are located inside organs, are covered by the epithelium, which is composed of a similar type of squamous or pseudo-squamous epithelial cells and protect these organs and our body. The basal cells of the squamous epithelium express keratin 5 (K5), K14, and p63 and are able to proliferate and differentiate in response to various stimuli, including growth factors, cytokines, chemokines, inflammatory irritants, and chemical carcinogens. These basal cells are also able to give rise to benign papilloma and malignant SCC. Deletions and reduction of CHUK, which encodes IKKα, reportedly promote SCC development in the skin, lungs, oral cavity, esophagus, and nasopharynx of humans [19,22,23,35,36,37,38,39,40].

#### 2.1.1. Skin Diseases and Cutaneous SCC

***IKK****α **activity in mouse embryonic development***. The skin, which is made up of the epidermis and dermis, is the largest organ. The epidermis consists of undifferentiated to terminally differentiated keratinocytes (a type of squamous epithelial cells) that form the cornified layer and protects our bodies. *Ikkα^−/−^* newborns have a strikingly thick epidermis that lacks the terminally differentiated keratinocytes and has significantly increased numbers of K5/K14- and Ki67-positive cells. As a result, bodily fluid flows out of *Ikkα^−/−^* skin, which causes the mutant mice to die [11]. Reintroducing IKKα in vivo and in vitro rescues the undifferentiation defect in *Ikkα^−/−^* mouse keratinocytes and the mutant mice [41]. Meanwhile, the fetal liver is a major target for IKKβ, IKKγ, and p65 during embryonic development [14,15,17]; whereas LPS injection induces a comparable level of the IKK kinase activity in the liver of *Ikkα^−/−^* and wild-type (WT) newborn pups [11]. The 32 and 36 serine residues of IκBα are used as a kinase substrate, analyzed by an immunoprecipitated kinase assay with an anti-IKKγ antibody. *Ikkα^−/−^* embryonic fibroblasts remain at a similar or slightly decreased NF-κB and IKK activity level in response to TNFα or IL-1β stimulation compared to WT cells, as detected with an anti-IKKβ antibody immunoprecipitated kinase assay [11]. Of interest, an anti-IKKγ antibody immunoprecipitated kinase assay showed slightly higher kinase activity in *Ikkα^−/−^* fibroblasts than in WT cells in response to IL-1β but not TNFα stimulation [41]. In addition, the IKK kinase and NF-κB activities increase in response to these stimuli in primary cultured *Ikkα^−/−^* keratinocytes compared to WT. Collectively, IKKα deletion is not able to fully block the IKK kinase and NF-κB activities compared to IKKβ or IKKγ deletion, or else IKKα deletion elevates IKK/NF-κB activity, depending on cell type.

***The importance of CHUK/IKK****α **in human embryonic development***. A *CHUK* nonsense mutation that creates a stop code at amino acid 422 to generate a truncated and unstable protein, which in turn causes IKKα loss, is identified in an autosomal recessive lethal syndrome [29]. The appearance of the mutant human fetuses at 12 to 13 weeks old, which is characterized by multiple defects in the face, limbs, kidneys, heart, skull bones, and lungs, is similar to that of the *Ikkα^−/−^* newborn pups (Table 1). Thus, CHUK/IKKα is essential for human embryonic development. On the other hand, humans with a *IKBKB* gene mutation undergo the normal embryonic development but suffer severe bacterial, viral, and fungal infections after birth [30,31,32,33]. Thus, IKKα is required for human and mouse embryonic development independently of TNFR1, while IKKβ and IKKγ are essential for protecting liver cells of mouse embryos from apoptosis through a TNFR1-led NF-κB pathway (Table 1).

***IKK****α **Transgene versus Ikk**α **gene deletion******in skin inflammation and tumorigenesis***. Of note, Tg-K5.mIκBα transgenic mice, in which there is a super suppressor mIκBα with mutations at amino acids 32 and 36 from serine to alanine, develop spontaneous skin SCCs associated with increased inflammation (Table 1) [20]. Ablation of the *Tnfr1* gene abolishes skin inflammation and SCC in Tg-K5. mIκBα;*Tnfr1^−/−^* mice [21]. Thus, it has been suggested that decreased NF-κB activity promotes skin tumorigenesis. However, Tg-K5.IKKβ mice show epidermal hyperplasia and oral tumors (Table 1) [25,26], and Tg-EDL2.IKKβ mice that overexpress IKKβ in the esophageal epithelial cells also develop hyperplasia in the esophagus [27]. On the other hand, Tg-K5.IKKα mice develop normal skin and esophagus at one day and one year of age [24]. Thus, the role of IKK and NF-κB in skin homeostasis and tumorigenesis is intriguing.

*Ikkα^+/−^* mice, which look normal, develop twice as many papillomas and 10 times as many malignant SCCs than *Ikkα^+/+^* mice in the chemical carcinogen–induced carcinogenesis setting [19] (Table 1). These *Ikkα^+/−^* SCCs lost the WT *Ikkα* allele, and IKK kinase activity is increased compared to *Ikkα^+/+^* carcinomas, which is correlated with increased ERK activity, poorly differentiated cell features, and WT *Ikkα* allele loss [19], indicating that IKKα loss promotes the malignant conversion from papillomas to carcinomas. Moreover, the expression of IL-1α, TNFα, TGFα, EGF, amphiregulin, HB-EGF, FGF2, FGF3, and VEGFA is higher in *Ikkα^+/−^* skin than in *Ikkα^+/+^* skin, treated with a chemical skin-tumor-promoter (inflammatory irritant). Although the chemical carcinogen stimulates IKKα expression in the normal skin and early benign tumors [19,42], the heterozygous *Ikkα* gene (*Ikkα^+/−^*) expresses a half dose of induced IKKα in response to stimuli compared to WT (*Ikkα^+/+^*), which attenuates IKKα anti-tumor activity. Thus, IKKα is a tumor suppressor with a haplo-insufficient feature.

To demonstrate whether IKKα loss induces spontaneous skin tumors, we ablated *Ikkα* with K5.Cre and found that *Ikkα^f/f^*;K5.Cre mice die within three weeks after birth [37] (Table 2). Furthermore, the inducible ablation of *Ikkα* in the basal keratinocytes or stem cells of adult *Ikkα^f/f^* mice by inducible K5.Cre^ER^ or K15.Cre^RP1^ with tamoxifen or RU486 results in spontaneous skin papillomas and SCCs [37]. Thus, IKKα deletion is a tumor driver. The tumor microenvironment modulates tumorigenesis. We have shown that inflammatory cytokines downregulate IKKα levels [23]. IKKα inactivation and increased macrophage numbers upregulate the expression of cytokines and chemokines, which are targets of NF-κB [43,44,45,46], and result in spontaneous lung SCCs, although the underlying mechanism requires further investigation.

On the other hand, IKKα overexpression inhibited chemical carcinogen–induced skin SCC metastasis or UVB-mediated skin carcinogenesis in transgenic Tg-Lori.IKKα or Tg-K5.IKKα mice, in which the IKKα cDNA is controlled by the loricrin promoter or the keratin 5 promoter [24,52]. Furthermore, the hyperproliferative epidermis lacking IKKα expressed increased IL-1, IL-6, and TNFα associated with increased macrophage infiltration. In contrast, overexpressed IKKα does not activate IKK kinase or NF-κB activity in the skin of Tg-K5.IKKα mice [53]; instead, it represses *Ikkα* deletion–mediated cytokine expression and macrophage infiltration in the skin of *Ikkα^f/f^*;K5.Cre;K5-IKKα mice. Overall, IKKα represses inducible NF-κB–related inflammation in skin.

***Neonatal skin manifestations versus tumorigenesis***. Intriguingly, all *Ikkβ^f/f^*;K14.Cre, *Ikkγ^f/f^*;K14.Cre, and *Ikkα^f/f^*;K5.Cre (and *Ikkα^f/f^*;K14.Cre) newborn pups specifically lacking either *Ikkβ*, *Ikkγ*, or *Ikkα* in skin keratinocytes look normal [37,47,48]. However, these mutant mice gradually develop severe skin inflammation and epidermal hyperplasia after three to four days and die within two to three weeks after birth (Table 2). Again, *Tnfr1* deletion rescues the skin and death phenotypes of *Ikkβ^f/f^*;K14.Cre and *Ikkγ^f/f^*;K14.Cre mice but it does not do so for *Ikkα^f/f^*;K5.Cre and *Ikkα^f/f^*;K14.Cre mice [37,47,48]. In addition, the epidermal layer is thicker in newborn mice than in adult mice, and the newborn skin has shorter hair follicles than adult mouse skin. The microbiome is closely associated with hair follicles and skin [37]. Thus, it is possible that alterations in IKKβ, IKKγ, or IKKα expression alter microbiota, which may contribute to epidermal hyperplasia and skin inflammation phenotypes in *Ikkβ^f/f^*;K14.Cre, *Ikkγ^f/f^*;K14.Cre, and *Ikkα^f/f^*;K5.Cre neonatal mice. Furthermore, inducible ablation of either *Ikkβ*, *Ikkγ*, or *Ikkα* in the skin of adult mice may have less of an effect on skin microbiota than in neonatal skin. Of note, *Iκbα^f/f^*;K5.Cre and *Iκbα^−/−^* mice show severe skin inflammation, whereas *p65(Rela)^f/f^*;K5.Cre mice develop normally [28,50,54] (Table 2). Taken together, it remains largely unclear whether and how IKK and NF-κB are involved in maintaining microbiota-related skin homeostasis and tumorigenesis.

Although *Tnfr1* knockout does not rescue *Ikkα^f/f^*;K5.Cre;*Tnfr1^−/−^* mice, the mutant skin highly express TNFα compared to WT [37]. Instead, EGFR reduction rescues a fraction of *Ikkα^f/f^*;K5.Cre;*Egfr^+/−^* mice characterized by hairlessness, suggesting that IKKα’s targets include EGFR during neonatal skin development. Inducible *Ikkα^f/f^*;K5.Cre^ER^ and *Ikkα^f/f^*;K15.Cre^RP1^ mice develop spontaneous skin tumor development, and treatment with GW2974, an EGFR inhibitor, prevents the tumorigenesis (Table 2). Inducible *Ikkγ* deletion in adult mice did not cause skin diseases, and inducible *Ikkβ* deletion induced skin inflammation in some mutant mice [48,49]. There are no skin tumors reported in mice with inducible *Ikkβ* or *Ikkγ* deletion in the skin. These analyses indicate that IKKα loss and reduction elevate the expression of NF-κB targets, such as TNFα and IL-1, which are correlated with skin inflammation and tumorigenesis. Furthermore, *Tnfr1^−/−^* and *Tnfa^−/−^* mice are resistant to chemical carcinogen–induced skin carcinogenesis compared to WT mice [55,56]. Although these cytokines, such as TNFα, are not required for mouse embryonic and skin development, these induced inflammatory cytokines can stimulate inflammation-associated carcinogenesis. Taken together, these results suggest that the different events take place at different stages of embryonic skin, neonatal skin, and skin tumor development. Many questions remain to be addressed.

***Impaired central tolerance-mediated autoinflammation***. The cause of skin inflammation and spontaneous tumors developed in Tg-K5.mIκBα mice remains a mystery, although *Tnfr1* deletion rescues the skin phenotypes in Tg-K5.mIκBα mice [20,21]. Over the past several years, we have learned that RANK/RANKL, CD40/CD40L, LTβR/Ltβ, IKKα, NIK, and RelB are required to develop medullar thymic epithelial cells (mTECs) [23,57,58,59,60,61,62,63,64]. The autoimmune regulator, which is expressed in mTEC, regulates the expression of tissue restrict antigens and other regulators that maintain the thymic microenvironment, which helps delete autoreactive T cells within the thymus and establish central tolerance [65]. Kinase-dead IKKα (IKKα-*KA/KA*), in which a lysine is replaced by an alanine at amino acid 44, an ATP binding site within the protein kinase domain, attenuates NF-κB (p65/p50) activity in mTEC and severely impairs mTEC and central tolerance development in *Ikkα^KA/KA^* mice (Table 2) [23]. The IKKβ and IKKγ levels in *Ikkα^KA/KA^* mTEC are decreased compared to WT. Different thymic stromal cells express distinct keratins, and mTEC expresses K5 [23]. Reintroduction of Tg-K5.IKKα partially elevates p65/p50 activity, rescues mTEC development, and dampens autoimmune phenotypes in *Ikkα^KA/KA^*;Tg-K5.IKKα mice compared to *Ikkα^KA/KA^* mTEC and *Ikkα^KA/KA^* mice. Therefore, a defect in IKKα and NF-κB pathways results in negative T-cell selection, central tolerance defects, and autoimmune diseases. *Ikkα^KA/KA^* newborn mice look normal. After three months, the mutants start to show autoimmune phenotypes, and with increasing age, the phenotypes become worse.

To date, mice lacking *Rank*, *Rankl*, *Cd40*, *Cd40l*, *Ltβr*, *Ltβ*, or *Nik* develop autoimmunity, but none of these knockout mice develop spontaneous tumors [57,58,59,60]. Gradually decreased IKKα levels in epithelial cells are correlated with severe skin inflammation and tumorigenesis, as *Ikkα^KA/KA^* mice age [23,66]. We demonstrated that the autoreactive CD4 T cells initiate the skin autoinflammatory phenotypes with increased macrophage and neutrophil numbers, inflammasome activity (NLRP3), uric acid levels, and expression of CCL2, CXCR2, CXCL1, and IL-1β in *Ikkα^KA/KA^* skin compared to WT [66]. NLRP3 inhibition or CCL2/CXCR2 deletion rescued the skin phenotypes in the mutant mice. Although *Tnfr1* deletion does not rescue the skin and death phenotypes in *Ikkα^f/f^*;K5.Cre mice [37], its knockout improves *Ikkα^KA/KA^* skin phenotypes (unpublished data), suggesting that an overexpressed TNF/TNFR1 pathway may be involved in the skin phenotypes of adult *Ikkα^KA/KA^* mice but is not a cause for *Ikkα* ablation-initiated embryonic and neonatal skin phenotypes.

Moreover, an overexpressed K5.IKKα transgene partially regulates mTEC and central tolerance development [23,67]. Most likely, the biological impact of the specific K5 promoter-driven transgenic gene is earlier than the function of the K5 promoter-driven Cre on the central tolerance establishment during embryonic thymus development because the specific Cre takes two steps and the transgenic gene only takes one step to execute its biological activities. Thus, Tg-K5.mIκBα mice may show autoinflammation-associated skin tumors because overexpressed mIκBα may downregulate NF-κB activity in mTEC [23], but *IκBα^f/f^*;K5.Cre mice may not develop the autoinflammatory phenotypes [50]. Furthermore, our unpublished finding showed that IKKα-KA/KA binds to IκBα more strongly than WT IKKα does. We then hypothesize that kinase-inactivated IKKα and unphosphorylated mIκBα enhance their interaction in the cytosol, which may ameliorate nuclear IKKα levels that regulate cell differentiation, proliferation, and tumorigenesis, and that overexpressed mIκBα may block IKKα anti-tumor activity. The K5 promoter is a very strong promoter to highly express the transgene in mice [24]. Thus, overexpression of mIκBα may have additional events beyond NF-κB pathways. Moreover, the overexpressed mIκBα has been shown to promote the development of SCC derived from activated *HRAS* SCC cells [68]. In contrast, overexpressed p65 or p50 increase cell senescence, suggesting that increased NF-κB inhibits skin SCC development, while decreased NF-κB promotes skin SCC development by regulating cell senescence [68]. The same mutations in the *IκBα* gene, which encodes IκBα, may not appear in human cancers (TCGA database). In general, NF-κB transcription factors regulate the expression of a broad spectrum of cytokines and chemokines that modulate tumor cell growth and survival and the tumor microenvironment. Some of IKKα’s targets share NF-κB’s pathways during inflammation and tumor development. We need to further reveal how IKKα regulates the expression of the inflammatory cytokines.

#### 2.1.2. Lung SCC, Esophageal SCC, and Nasopharyngeal Carcinoma

The basal cells in the internal surface of lung air tubes, esophagi, and nasopharynges, which express K5/K14 and p63, are squamous-like epithelial cells. Like keratinocytes, these cells can be induced to differentiate and proliferate as well as give rise to well-differentiated papillomas and undifferentiated malignant carcinomas. *Ikkα^KA/KA^* mice at six to 10 months of age on an FVB background develop spontaneous lung SCCs [22]. The IKKα levels in the lungs of *Ikkα^KA/KA^* newborn pups are similar to WT. From four to 16 weeks of age, the IKKα levels are gradually decreased, which is associated with lung SCC development [22]. Reintroduced transgenic IKKα prevents lung SCC development. Thus, IKKα reduction promotes lung carcinogenesis. The mouse *Ikkα^KA/KA^* lung SCCs express human lung SCC molecular traits, including reduced tumor suppressor p53 and Rb1, and overexpressed p63, Trim29, ROS1, Rhov, c-Myc, and stem cell markers. In addition, depleting macrophages prevents lung SCC development in this setting. Thus, the significantly increased number of infiltrating macrophages is essential for lung SCC development in *Ikkα^KA/KA^* mice. We detected increased expression of TNFα, IL-1β, IL-6, IL-4, IL-13, CCL2, CXCL5, CXCL11, and CXCL8 in *Ikkα^KA/KA^* lungs compared to WT lungs. These chemokines promote macrophage induction, migration, and recruitment [22,69,70]. Of note, these lung SCC and WT lungs express comparable p65 levels, suggesting that IKKα reduction does not downregulate NF-κB expression, though it is associated with marked expression of multiple cytokines, chemokines, and macrophage infiltration during lung SCC development.

The esophagus is one organ of the gastrointestinal tract, and it frequently has contact with environmental infectious agents. The immune system prevents bacterial and fungal colonization and expansion. Of interest, most esophageal SCCs (ESCCs) are found in China and South Asia, where the weather is humid and damp. Such an environment can foster fungal growth and contaminate food. We found that these human ESCCs are associated with increased fungal infection [23]. Defects in immunity or epithelial cells allow bacterial and fungal colonization in the oral and esophageal organs, promoting disease pathogenesis. *Ikkα^KA/KA^* mice not only develop autoinflammation but show downregulated IL-17 expression in T cells in response to fungal stimulation [23]. IL-17 fights against fungal infection [71,72]. C57BL6 *Ikkα^KA/KA^* mice showed increased fungal colonization in the oral cavity and esophagi, and approximatively 20% of five-month-old *Ikkα^KA/KA^* mice develop spontaneous ESCC. Oral fungal inoculations promote ESCC incidence, while anti-fungal drug treatment decreases ESCC development in *Ikkα^KA/KA^* mice [23]. Thus, increased fungal infection promotes ESCC development. These mouse ESCCs expressed reduced IKKα, p53, and p16 but increased EGFR activity and p63, which are the hallmarks for human ESCCs. They also express increased NF-κB1 and many cytokines and chemokines, including CCL7, CCL8, and IL-23, with infiltrating macrophages that express increased PD-L1, CXCL11, TNFα, and IL-6 [23]. Anti-fungal drug treatment decreased in infiltrating macrophage numbers and carcinogenesis. Overall, infection-mediated inflammation promotes esophageal carcinogenesis.

Nasopharyngeal carcinoma (NPC), which frequently occurs in Southeast Asia, is a deadly disease, because more than 95% of patients with NPC have type III undifferentiated carcinomas at first diagnosis [40]. NPC arises from the pharyngeal mucosal space and is associated with Epstein-Barr virus infection in most cases. Downregulated CHUK/IKKα expression is significantly correlated with aggressive and undifferentiated NPCs that express increased K13 and decreased vimentin [40]. IKKα deletion in human NPC cells promotes tumorigenesis, but reintroduced IKKα inhibits tumorigenesis derived from human NPC cells. EZH2 is identified as an inhibitor for downregulating IKKα expression via increasing the methylation on the *Ikkα* promoter. Also, decreased IKKα expression is correlated with increased ERK activity and expression of VEGF and MMP9 in these human NPCs. Whether IKKα reduction modulates the tumor microenvironment requires further investigation.

### 2.2. Adenocarcinoma

ADC is a type of cancer that originates from lung, pancreas, colon, prostate, or esophagus glands. The Cancer Genome Atlas (TCGA) database reveals activated *KRAS* mutations in approximately 35% of human lung ADCs, 90% of pancreatic ductal adenocarcinomas (PDACs), and 45% of colorectal ADCs (cBioPortal, *Nature* 2014, *Nature* 2016, and PanCancer Atlas). A high rate for activated *KRAS* mutations is a common feature for these different human ADCs.

#### 2.2.1. Lung ADC

Lung ADC is derived from lung type II alveolar epithelial cells. *CHUK* nonsense mutations and homozygous/hemizygous deletions were found in human lung ADCs (cBioPortal, TCGA, *Nature* 2014) [38]. Patients with lung ADCs carrying double *CHUK* hemizygous deletions and *KRAS* mutations have significantly decreased survival rates compared to patients with *KRAS* mutations alone. *Ikkα* deletion specific in the lungs, induced by intratracheally injected adenovirus-Cre, causes spontaneous lung ADC development and promotes Kras^G12D^-initiated lung ADC development in *Kras^G12D^*;*Ikkα^ΔLU^* (*Ikkα* deletion in the lungs) mice (Table 3). Although the levels of NF-κB components are comparable in *Kras^G12D^* lung ADC and *Kras^G12D^*;*Ikkα^ΔLU^* ADCs, IKKα reduction in the lung ADCs elevated the expression of inflammatory cytokines and chemokines, including NF-κB targets [38]. Reintroducing IKKα into ADC cells suppressed tumor incidence and burden derived from the ADC cells in the lungs and repressed the expression of multiple cytokines, which indicates that impaired IKKα expression in cancer cells regulates the tumor microenvironment.

In addition, specifically induced *Ikkα* ablation in lung type II epithelial cells by Sftpc-CreER^T2^ promotes a carcinogen-initiated lung cancer development, but *Ikk*β deletion inhibits lung ADC development in this setting (Table 3) [73]. We, like Chavdoula and colleagues, consistently demonstrated that IKKα deletion promotes the development of lung ADCs derived from a human ADC cell line, while reintroduced IKKα inhibits them [73]. Of note, p52 reduction inhibits lung tumorigenesis in the same setting [73]. These results suggest that NF-κB contributes to lung ADC development and that, during tumorigenesis, IKKα and NF-κB may function in two parallel pathways. Thus, the intriguing interplay between NF-κB and IKKα should be further investigated. Moreover, IKKβ depletion or NF-κB reduction inhibits lung epithelial cell proliferation and Kras^G12D^-mediated lung ADC development [75,76]. In contrast, another study reported an opposite finding in which deletion of *Ikkα* but not *Ikkβ* reduces Kras^G12D^-mediated lung ADC development through NF-κB activity [74], which is not supported by human TCGA data analyses [38].

#### 2.2.2. Pancreatic ADC and Colorectal Carcinoma

Furthermore, patients with PADC carrying *CHUK* mutations have decreased survival rates (cBioPortal, Pancreas). *Ikkα* deletion in pancreatic cells mediated by Pdx1-Cre induces pancreatitis associated with the increased expression of TNFα, IL-1β, IL-6, MCP-1, and RANTES, which are NF-κB’s targets [77]. Patients with pancreatitis have increased incidence of pancreatic cancer. In addition, *Ikkα* deletion significantly promotes Kras^G12D^-innitiated PDAC development in mice (Table 3) [39]. Thus, IKKα reduction in pancreatic cells promotes inflammation associated with NF-κB targets.

Activated *KRAS* mutations are found in 40–50% of human colorectal carcinomas (cBioPortal, TCGA, Colorectal Adenocarcinoma cohorts). Meanwhile, the gut microbiota regulates carcinogenesis. In mouse models, chemical irritators and carcinogens have been broadly used for studying inflammation-associated colitis and carcinoma development. In this setting, *Ikkα* deletion in the intestinal epithelial cell (IEC) increases *Citrobacter rodentium* infection, and associated inflammation promotes DSS-mediated colitis [78]. *Ikkα* deletion in IEC cells recruits type III innate lymphocytes by elevating thymic stromal lymphopoietin to downregulate IL-22 in *Ikkα^ΔIEC^* mice. On the other hand, *Ikkβ* inactivation in IEC cells reduces colitis, which is the opposite of *Ikkα* ablation. This indicates IKKα and IKKβ have a distinct role in inflammation formation and colitis development [78]. Thus, IKKα prevents induced inflammation and is required for intestine-colon homeostasis in response to infectious and inflammatory stimuli. Interestingly, *Nik* deletion, as well as *Ltbr* deletion, increased inflammation in the colon following DSS treatment [79]. Therefore, the relationship of NIK, IKKα, and NF-κB in this setting needs further investigation.

### 2.3. Breast Cancer and Prostate Cancer

Breast and prostate cancers are related to hormone alterations. Interestingly, TCGA (cBioPortal, METABRIC, Nature 2012 & Nat Commun 2016) data analyses show that a proportion of these human cancers show increased or amplified cyclin D1, which is encoded by the CCND1 oncogenic gene. RANKL and RANK, the TNFR family members, regulate mammary gland development [80]. Consistently, kinase-inactive *Ikkα* knock-in (*Ikkα^AA^*) mice, in which two serine residues were replaced by alanine residues at amino acid 177 and 181, show a defect in mammary gland development [34]. *Ikkα^AA^* mammary epithelial lobuloalveolar cells reduced growth and NF-κB activity in response to RANKL but not TNFα treatment in a culture system [34]. Mammary tumorigenesis is inhibited in *Ikkα^AA^*;Tg-MMTV.cyclin D1 mice as well as in Tg-MMTV.mIκBα mice compared to Tg-MMTV.cyclin D1 mice [34]. Thus, NF-κB is important for breast tumor development through RANKL/RANK/IKKα to the oncogene cyclin D1. Also, RANK/RANKL is involved in human breast cancer and has been used for therapeutic targets. The kinase-dead *Ikkα* knock-in (*Ikkα^KA/KA^*) mice develop a more severe defect in the mammary gland compared to *Ikkα^AA^* mice (unpublished data). In addition, the *Ikkα^KA/KA^* female mice can have a single pregnancy with one or two babies and no milk production, and then these females because infertile (unpublished data). Whether an autoimmune condition contributes to this defect in reproductive organ development in *Ikkα^KA/KA^* mice remains to be investigated [23]. Overall, *Ikkα^AA^* mice and *Ikkα^KA/KA^* mice share a common defect in mammary gland development, although these *Ikkα^KA/KA^* mice develop additional spontaneous lung and skin SCC associated with IKKα reduction. Based on these results, the RANKL/RANK/IKKα pathway functions as a tumor promoter for the mammary gland cancer development.

TRAMP mice that overexpress SV40 in the prostate organ develop spontaneous prostate tumors [51]. The prostate tumorigenesis and metastasis were inhibited, which correlated with decreased expression of multiple cytokines, in *Ikkα^AA^*;TRAMP male mice through a NF-κB RANK/ANKL pathway to regulate the expression of Maspin that is a tumor suppressor, compared to TRAMP transgenic mice [51]. This finding suggests that NF-κB may stimulate prostate cancer development. Interestingly, a PHLPP/FKBP51/IKKα complex has been reported to upregulate NF-κB and AKT activity in castration-resistant prostate cancer development [81], although the specific role of IKKα in this complex needs to be determined. In the future, the effect of cell-specific *Ikkα* deletion on breast and prostate cancer development should be investigated.

## 3. Conclusions

Overall, NF-κB and its targets intensify inflammation and accelerate SCC and ADC tumorigenesis. On the other hand, defective immunity-mediated systemic autoinflammation, initiated by germline IKK/NF-κB inactivation in the lymphoid organ, which cooperates with oncogenes or tumor suppressors, can also contribute to tumorigenesis. Thus, NF-κB can regulate tumorigenesis at different stages. IKKα reduction and ablation result in cutaneous, lung, oral, esophageal, nasopharyngeal, and pancreatic carcinogenesis associated with increased inflammation. IKKα inactivation–induced inflammation shows a similar pattern to that mediated by NF-κB activation. These findings will provide potential therapeutic targets for human cancer.

To date, most studies focus on IKKα, TNFR family members, and carcinogenesis. We still do not know about the crosstalk between IKKα-mediated pathways and Toll-like receptor pathways. Of note, the microbiota plays important roles in modulating inflammation and carcinogenesis. This may explain some contradictory results for IKK and NF-κB among different laboratories. In addition, we found that increased inflammation downregulates IKKα levels in tumors. Thus, the in vitro cultured cell lines may have higher IKKα levels than in vivo tumors. Furthermore, by western blotting, a non-specific band close beneath the IKKα band can mask a real IKKα protein band when IKKα levels are examined in mouse and human tissues, which may also contribute to the disagreements regarding IKKα expression among different laboratories.

## Figures and Tables

**Table 1:** Comparison of physiological activities and IKK/NF-κB activities in genetically modified mice and humans.

Mice	Developmental Phenotypes	*Tnfr1* KO/NF-κB	Tumorigenesis	References
*Ikk* *α^−/−^*	Die soon after birth, marked epidermal hyperplasia	*Tnfr1* KO does not rescue mutants; slightly reduced or increased NF-κB activity	*Ikkα^+/−^* mice develop carcinogen-induced skin carcinogenesis	[11,18,19]
*Ikk* *β^−/−^*	Embryonic lethality, liver cell apoptosis, hemorrhage	*Tnfr1* KO rescues mutants, blocks NF-κB activity	Not tested	[15,16]
*Ikk* *γ^−/−^*	Embryonic lethality, liver cell apoptosis, hemorrhage	*Tnfr1* KO rescues mutants, blocks NF-κB activity	Not tested	[13,14]
*p65(Rela)^−/−^*	Embryonic lethality, liver cell apoptosis, hemorrhage	*Tnfr1* KO rescues mutants, blocks NF-κB activity	Not tested	[12,17]
Tg-K5.mIκBα	Normal embryonic development; skin phenotypes with cell apoptosis and inflammation	*Tnfr1* KO rescues mutants	Spontaneous skin tumors	[20,21]
Tg-K5.IKKα	Normal mice and normal skin from one day to more than one year	Not tested for *Tnfr1* KO; no increase in skin NF-κB	Inhibit UVB-induced skin carcinogenesis; rescue lung squamous cell carcinomas and autoimmunity	[22,23,24]
Tg-IKKβ	Normal embryonic development	Not tested for *Tnfr1* KO; increased NF-κB activity	Epidermal hyperplasia and spontaneous oral tumors	[25,26]
Tg-EDL2.IKKβ	Normal embryonic development	Not tested for *Tnfr1* KO; increased NF-κB activity	Esophageal hyperplasia	[27]
*I* *κb* *α^−/−^*	Die within 6 weeks after birth with severe skin inflammation	Not tested for *Tnfr1* KO; increased NF-κB activity	Not tested	[28]
Human CHUK mutations	Mutations at amino acid 422 to generate a stop code; embryonic lethality at 12–14 weeks	Appearance like *Ikkα^−/−^* mice; defects in the face, limbs, kidneys, heart, lungs, bone	Gene mutations and deletions associated with reduced survival of lung adenocarcinoma patients	[29]
Human IKBKB mutations	Infants and children with *IKBKB* mutations show severe infection	Reduced NF-κB activity	Detected mutations but not known for lung cancer survival	[30,31,32,33]

**Table 2:** Comparison of physiological activities and tumorigenesis in mice lacking IKK/NF-kB in specific tissues.

Mice	Neonatal Phenotypes	*Tnfr1* KO/NF-κB	Tumorigenesis	References
*Ikkα^f/f^*;K5.Cre	Die within 3 weeks; neonatal epidermal hyperplasia and severe inflammation	*Tnfr1* KO does not rescue mutants; increased TNF and NF-κB	NA	[37]
*Ikkα^f/f^*;K14.Cre	Die within 3 weeks; neonatal epidermal hyperplasia and severe inflammation	Not rescued	NA	[37]
*Ikkβ^f/f^*;K14.Cre	Die within 3 weeks; neonatal epidermal hyperplasia and severe inflammation	*Tnfr1* KO rescues mutants and skin lesions	NA	[47]
*Ikkγ^f/f^*;K14.Cre	Die within 3 weeks; neonatal epidermal hyperplasia and severe inflammation	*Tnfr1* KO rescues mutants and skin lesions	NA	[48]
*Ikkα^f/f^*;K5.Cre^ER^	Skin inflammation, hairless, and spontaneous skin tumors	Not tested for *Tnfr1* KO; *Egfr^+/−^* and EGFR inhibitor rescue	*Egfr^+/−^* and EGFR inhibitor prevent skin tumorigenesis	[37]
*Ikkα^f/f^*;K15.Cre^RP1^	Skin inflammation, hairless, and spontaneous skin tumors	Not tested for *Tnfr1* KO	EGFR inhibitor prevents skin tumorigenesis	[37]
*Ikkβ^f/f^*;K14.Cre^ER^	Skin inflammation in some mice at 3⎯6 months of age	Anti-TNFR1 antibody rescues skin lesions	No skin tumors reported	[49]
*Ikkγ^f/f^*;K14.Cre^ER^	No severe skin phenotypes	Not tested for *Tnfr1* KO	No skin tumors reported	[48]
*p65(Rela)^f/f^*;K5.Cre	No severe skin phenotypes	Not tested for *Tnfr1* KO	No skin tumors reported	[50]
*Iκbα^f/f^*;K5.Cre	Skin inflammation	*Tnf* KO/*LTβ* KO rescues skin phenotypes; increased NF-κB	No skin tumors reported	[50]
*Ikkα^AA/AA^*: mutations 177/181-Ser/Allan	Defects in mammary gland development through reducing RANKL/RANK, normal skin	Not tested for *Tnfr1* KO	Reduced breast and prostate cancer	[34,51]
*Ikkα^KA/KA^*: mutations 44-Lys/Allan	Defects in central tolerance, autoimmunity, and mammary glands	Unpublished data: *Tnfr1* KO partially reduces skin phenotypes	Spontaneous skin, lung, and esophageal tumors correlated with decreased IKKα levels	[22,23]

**Table 3:** Impact of IKK/NF-κB activities in lung and pancreatic adenocarcinomas (ADCs).

Mice	Tumorigenesis	NF-κB activity	References
*Ikkα^f/f^*;Ad.Cre-intratracheal	Spontaneous lung ADC; promoted Kras^G12D^-mediated lung ADC	Similar NF-κB activity in these ADCs from different groups	[38]
*Ikkα^f/f^*;Sftpc.Cre^ER^	Promoted carcinogen-induced lung ADC development	p52 reduction inhibited ADC development derived from human ADC cells	[73]
*Ikkα^f/f^*;Sftpc.Cre or Scgb1a1.Cre	Inhibited Kras^G12D^-mediated lung ADC development	Reduced NF-κB activity	[74]
*Ikkα^f/f^*;Pdx-1.Cre	Promoted Kras^G12D^-mediated pancreatic carcinoma	Not tested	[39]
IKKβ depletion	Inhibited lung epithelial proliferation and Kras^G12D^-mediated lung ADCs	Inhibited NF-κB activity	[75,76]

## Data Availability

Not applicable.
